# Comparison between Discrete Multi-Wavelength Near-Infrared Spectroscopy and Bioelectrical Impedance Analysis in the Assessment of Muscle Mass for Community-Dwelling Older People

**DOI:** 10.3390/jcm13082350

**Published:** 2024-04-18

**Authors:** Jinyoung Shin, Eunki Park

**Affiliations:** 1 Department of Family Medicine, Konkuk University Medical Center, Konkuk University School of Medicine, Seoul 05030, Republic of Korea; 2 Yonsei Joy Medical Clinic, Jungnang-gu, Seoul 05030, Republic of Korea

**Keywords:** muscle, investigative techniques, discrete multi-wavelength, near-infrared spectroscopy

## Abstract

(1) **Background**: This study evaluated the clinical implications of a new measurement technique for muscle mass using discrete multi-wavelength near-infrared spectroscopy (DMW-NIRS) compared with multifrequency bioelectrical impedance analysis (BIA) in older adults. (2) **Methods**: In a cross-sectional study involving 91 participants aged 65 years, the agreement of total lean mass for each measurement was assessed using the intraclass correlation coefficient (ICC) and Pearson’s correlation analysis. The study was conducted at a university hospital from 10 July 2023 to 1 November 2023. (3) **Results**: A total of 45 men (mean age, 74.1) and 46 women (mean age, 73.6) were analyzed. In the comparisons of total lean mass between DMW-NIRS and BIA, ICC (2.1) was 0.943 and Cronbach’s α coefficient was 0.949 (*p* < 0.001). Across all segments of lean mass, we found excellent agreement with the ICCs (>0.90) and acceptable values of the correlation coefficients (>0.6) between DMW-NIRS and BIA. (4) **Conclusions**: This study confirmed agreement in the measurements of muscle mass between portable devices using DMW-NIRS and BIA among community-dwelling older adults. A simple screening of muscle mass in a home setting would help to detect early decreases in muscle mass.

## 1. Introduction

The involuntary loss of muscle mass in older adults is accompanied by a normal aging process. It has been reported that muscle mass decreases by approximately 1% and muscle strength decreases by 2.5~3% annually starting from the age of 60 [1]. However, the progressive and generalized degradation of skeletal muscle is now recognized as a disease called sarcopenia, which is associated with adverse health outcomes, including increased morbidity, mortality, falls, hospitalization, and poor health-related quality of life [1,2,3,4]. Patients with sarcopenia have increased medical costs during hospitalization, regardless of whether they are younger or older than 65 years [5].

Sarcopenia has been overlooked and undertreated in mainstream practice, apparently because of the complexity of determining what variables to measure, how to measure them, what cutoff points best guide diagnosis and treatment, and how to best evaluate the effects of therapeutic interventions [6]. Current clinical guidelines for the International Clinical Practice Guidelines for Sarcopenia (ICFSR) recommend annual screening for muscle mass reduction in individuals aged ≥65 years or after the occurrence of health events, such as hospitalization [7]. According to a literature review, the most common methods for measuring muscle mass are dual-energy X-ray absorptiometry (DEXA; 43.6%), bioelectrical impedance analysis (BIA; 19.3%), computed tomography (CT; 25.6%), and others (11.5%) [8]. BIA is relatively inexpensive, portable, requires minimal training in its use, and presents no health risks to volunteers [9]. Based on previous studies, multifrequency BIA may provide a comprehensive and valid approach to body composition assessments [9,10]. BIA, which is relatively easy to use, is endorsed by both the European Working Group on Sarcopenia in Older People [4] and the Asian Working Group for Sarcopenia [11]. Nevertheless, several studies have reported that multifrequency BIA tends to underestimate fat mass and percentage of body fat compared to DEXA in some populations [9,12,13,14]. BIA is affected by posture; for example, in bedridden patients, the accuracy depends on the position of the electrodes, necessitating high-cost specialized equipment [15]. Additionally, in individuals with severe obesity (body mass index [BMI] > 35), appendicular lean mass tends to be overestimated compared with DEXA [16]. There has been a lack of studies on simple devices to measure muscle mass in older people whenever they want at a low cost, without their needing to visit hospitals, such as devices that can be used in homes or residential facilities.

A portable, discrete, multi-wavelength, near-infrared spectroscopy (DMW-NIRS) device is positioned on the skin, illuminates the skeletal muscle with near-infrared light, and detects the light that is reflected through it as a consequence of the amount of light absorbed by the tissue, making it a valid, reliable, and inexpensive wireless instrument in real-time [17]. Technically, it can measure variables such as water content, lipids, oxy-hemoglobin (HbO_2_), and deoxyhemoglobin (HHb), as well as other derivatives, such as total hemoglobin concentration (THC = HbO_2_ + HHb) and muscle oxygen saturation, expressed as a percentage (StO_2_ = HbO_2_/THC) [17]. From these values and age, sex, and BMI, the model-predicted lean muscle mass was determined. A new portable device such as Fitto^®^ provides real-time muscle mass measurements using the aforementioned variables. However, it has not yet been revealed whether these measurements are valid compared to existing muscle measurement indicators in the elderly.

Therefore, we compared DMW-NIRS with BIA to assess muscle mass in community-dwelling older adults in home settings.

## 2. Materials and Methods

### 2.1. Study Design

This study was designed as a cross-sectional study. The primary endpoint was agreement between the total lean mass of Fitto^®^ (Olive Healthcare, Seoul, Republic of Korea) and InBody 770^©^ (Biospace, Seoul, Republic of Korea), and the secondary endpoint was reliable measurements of muscle mass in a home setting, which recruited 91 elderly participants and was conducted in an outpatient clinic at a university hospital in Seoul, Korea, from 10 July 2023 to 1 November 2023. The study protocol was established according to the guidelines of the Declaration of Helsinki and was approved by the Institutional Review Board (IRB file no. KUMC 2022-11-033, 28 April 2023). When considering a correlation efficiency of 0.7 as acceptable for convergent validity analysis, and reflecting the binomial test, in one sample case, with a constant proportion of 0.5, effect size of 0.2, alpha value of 0.05, and power of 0.85, the target sample size for both males and females was calculated to be 45 individuals each.

### 2.2. Study Participants

This study recruited participants aged 65 years and above who had physical performance capabilities that did not require assistance in daily activities and were willing to participate in a Senior Community Center. The volunteers were evaluated after obtaining signed informed consent. Participants were excluded from the study if they had a malignancy, acute stroke, or dementia that was being treated or was not being controlled. The following individuals were excluded from this study: vulnerable subjects, those with measurement difficulties due to surgical procedures or tattoos/moles, and individuals with implanted electronic medical devices such as pacemakers. 

The participants were surveyed for age, sex, and comorbidities. We asked whether they had difficulties in their daily life because of low vision/hearing impairment, to which they responded “yes” or “no” to confirm visual and auditory impairments. We examined height and weight and calculated BMI as weight divided by height squared. Waist circumference was recorded as the measurement at the thinnest point. All surveys and measurements were conducted by a trained researcher. We collected data on adverse effects related to the device measurements. 

### 2.3. Muscle Mass Measurement

We examined the variables, such as HbO_2_, HHb, THC, and oxygen saturation StO_2_, of the 21 areas (both lateral deltoids, biceps brachii, pectoralis major, forearm muscles, triceps brachii, erector spinae, quadriceps femoris, lateral gastrocnemius, hamstrings, and rectus abdominis, and external obliques) by DMW-NIRS named Fitto^®^ (Olive Healthcare, Seoul, Republic of Korea) with standing posture, after obtaining written consent. The total time required for each measurement, which involved grounding for approximately 2–3 s, was less than 5 min. The total lean mass was calculated as the sum of the lean mass of both arms and legs, as well as the trunk. Muscle mass was calculated using the values obtained from the first measurement of each body part, which were measured twice to determine technician/user error and interobserver differences (e.g., electrode placement and body position). 

BIA has been proposed as a safe, fast, and noninvasive measurement of whole-body and fluid compartment composition [18]. BIA calculates fat-free mass using a two-compartment chemical model of body composition and mathematical equations [19,20]. BIA measurements were performed using an InBody 770^©^ (Biospace, Seoul, Republic of Korea) with multifrequency-based proprietary algorithms, according to the manufacturer’s instructions. It was measured in a standing posture with feet apart and elbows extended to avoid body contact for approximately one min. The bare feet made positive contact with the base electrodes at the heels and forefeet, and the subjects grasped two handle electrodes for direct contact, with two more electrodes for each hand at the thumbs and forefingers [21]. Calf circumference was recorded as the measurement taken at the thickest part of both calves using a nonelastic tape, which has a moderate to high sensitivity and specificity for predicting low skeletal muscle mass [22,23]. 

### 2.4. Statistical Analysis

The descriptive characteristics of the study participants are presented as the mean ± standard deviation (min–max) according to sex. The demographic and clinical characteristics were compared using independent t-tests for continuous variables. All analyses used logarithmically transformed muscle mass; however, the presented values of the measurements were not log-transformed. We calculated the mean values and standard deviations of lean mass in the total, right/left arm and leg, and trunk, and the differences according to the measurement methods, such as DMW-NIRS and BIA. We estimated the level of agreement between the experimental and reference conditions using the intraclass correlation coefficient (ICC). We assessed ICC using a two-way random model with a single measure (2,1), where values >0.75 were considered “good” and >0.90 “excellent” relative agreement [24]. The internal consistency of the instrument was evaluated using the Cronbach alpha coefficient, which indicates a good internal consistency of >0.7 (>0.5 for scales with less than five items) [25]. Pearson’s correlation analysis of lean mass values measured using DMW-NIRS and BIA was also performed. The strength of the correlation coefficients was categorized as weak (<0.4), moderate (0.4–0.69), or strong (≥0.7) [26]. Statistical analysis was performed using IBM SPSS (version 27.0; SPSS Inc., Chicago, IL, USA), and statistical significance was set at *p* < 0.05.

## 3. Results

A total of 91 participants (men: 45; women: 46) were enrolled (Table 1). The mean ages of the men and women were 74.1 and 73.6 years old, respectively. Age and BMI did not differ between men and women; however, increases in waist and calf circumferences were observed in men. More comorbidities were present in the men. Musculoskeletal diseases were more prevalent in women.

In the comparisons between the DMW-NIRS and BIA of the total and each part of the lean mass of the study participants, we found good consistency (ICC > 0.900, *p* < 0.001) (Table 2). The ICC values for the left and right sides of the upper and lower limbs were similar. 

**Figure 1:**
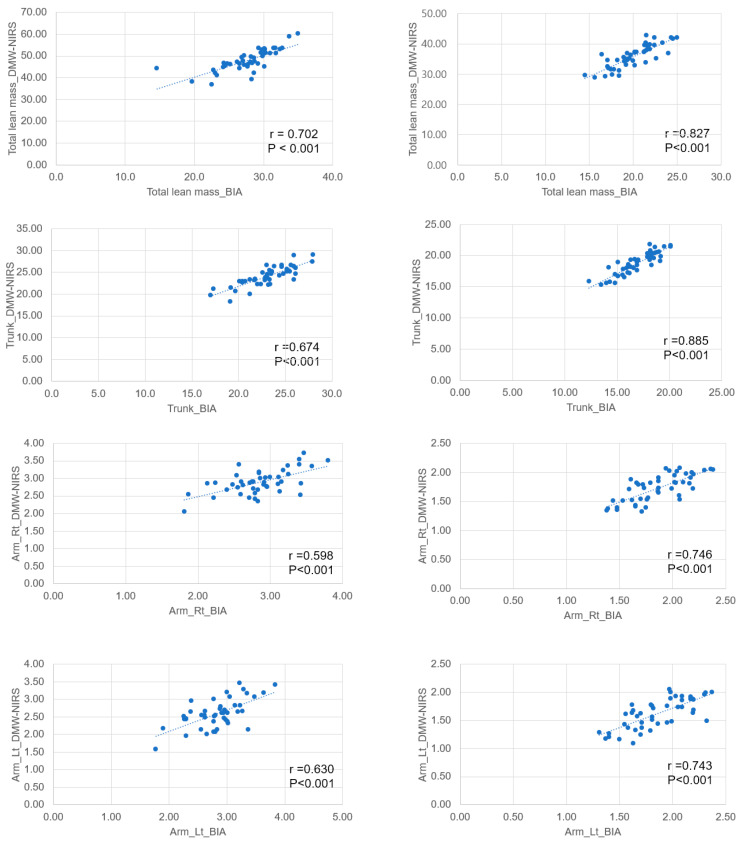

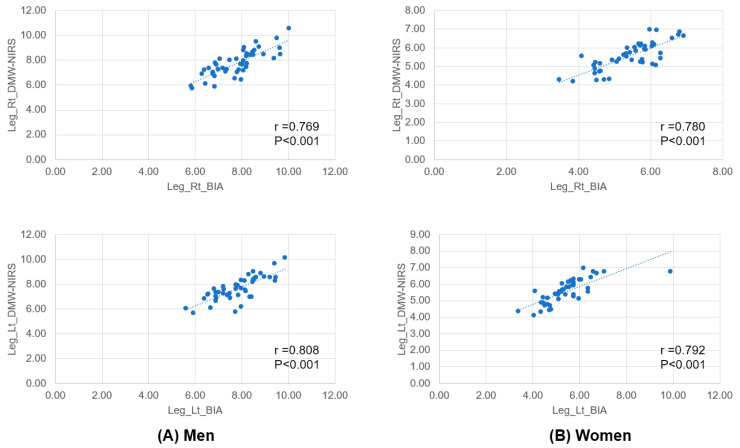
Comparison of near-infrared spectroscopy (DMW-NIRS) and bioelectrical impedance analysis (BIA) according to sex.

When comparing muscle mass measured by BIA and DMW-NIRS according to sex, we found a strong positive correlation (>0.7) between the two measurements, except for the upper arm in men (r = 0.598) (Figure 1). The correlation between both legs was similar in men and women. However, the other body parts showed higher correlations in women.

We found a correlation between calf circumference and lower limb muscle mass, as measured using DMW-NIRS in Table 3. Additionally, calf circumference was correlated with total muscle mass and upper-limb muscle mass measured using DMW-NIRS. In women, the correlation between calf circumference and lean mass was significant (*p* < 0.001), regardless of the body site.

## 4. Discussion

This study confirmed the agreement in the measurements of muscle mass between the portable devices DMW-NIRS and BIA among community-dwelling older adults. In the primary outcome of this study, the total lean mass measured using the two devices showed excellent agreement (β = 0.943). We confirmed the feasibility of measuring muscle mass in older adults using DMW-NIRS in comparison to valid tools such as BIA and calf circumference [27]. Furthermore, we confirmed the possibility of easily measuring muscle mass whenever needed, without visiting a hospital, because no well-validated and reliable tools are available for measuring muscle mass in a home setting [28]. A quick screening of muscle mass using a feasible device in a home setting would be beneficial for identifying the early stage of a decrease in muscle mass. 

A muscle mass evaluation was performed using different methods based on radiological images, biological measures (creatine dilution test), or anthropometric prediction equations [29]. The accuracy and reliability of these assessments mostly depend not only on the technical variances, but also on the time availability, radiation dose, costs, and patient involvement that must be considered in clinical use [29].

According to sex, no difference in agreement between DMW-NIRS and BIA was observed. Men and women had differences in body composition; men had a greater muscle mass than women; however, women had a greater fat mass. Therefore, BIA and calf circumference in men showed a higher correlation with conventional measurements of muscle mass than those in women [30]. However, DMW-NIRS measurements of muscle mass may be less influenced by fat mass than other measurements, with similar correlations based on sex. We found no significant errors or adverse effects in the measurements. 

The Asian Working Group for Sarcopenia 2019 recommended case findings using calf circumference (men < 34 cm, women < 33 cm) or the SARC-F questionnaire (≥4 points; strength, assistance in walking, rising from a chair, climbing stairs, and falls) to primary care physicians, followed by an assessment for diagnostic measurement using DEXA or BIA in a hospital setting [23]. DMW-NIRS is a measurement of muscle mass based on clinical implications to facilitate timely intervention in community healthcare and prevention settings before ‘sarcopenia’ or ‘sarcopenia’.

However, several limitations should be considered when interpreting the results. This study compared the muscle mass measured using DMW-NIRS with that measured using BIA as a gold standard, which is a transportable and executable device, in a systematic review of 62 studies [28]. However, BIA is dependent on individual characteristics, including edema and diuretics [31]. BIA estimates the lean mass from the total body water divided by the hydration coefficient based on the assumption that the hydration status is constant [13]. Therefore, it can overpredict fat-free mass in young men and women (mean age 30.4 ± 7.8 in men and 28.4 ± 7.0, respectively) [21]. If hydration status and total body weight are not constant because of disease or treatment, the results may be incorrect. Measuring limb muscle mass with DEXA also includes the skin, fat within the muscle, and connective tissue (fibrous tissue), which are considered drawbacks [32]. 

Considering that the study participants were of normal weight, differences in concordance may exist among elderly individuals who are underweight, overweight, or obesity [12,13]. As this study was conducted using single measurements, it is necessary to confirm the reliability of repeated measurements and the ability to detect changes in body composition.

## 5. Conclusions

The current study provides clinical evidence that can be used to examine muscle mass using DMW-NIRS as a valuable screening tool for older adults, particularly when using readily available and simple tools in home settings or residential facilities. Further studies are needed to determine the accuracy of muscle mass with clinically meaningful reference measurements, including muscle strength and physical performance, for use in older adults. 

## Figures and Tables

**Table 1:** Characteristics of participants (*N* = 91).

	Men (*N* = 45)	Women (*N* = 46)	*p*-Value
Age (years)	74.1 ± 5.0 (66–84)	73.6 ± 6.05 (66–87)	0.629
Height (cm)	166.1 ± 5.0 (155.5–175.1)	152.7 ± 6.4 (132.7–162.1)	<0.001
Body weight (kg)	67.5 ± 7.7 (50.0–83.9)	56.6 ± 7.8 (42.4–74.2)	<0.001
Body mass index (Kg/m^2^)	24.6 ± 2.5 (18.5–30.1)	24.2 ± 3.1 (18.6–32.2)	0.516
Waist circumference (cm)	83.0± 7.4 (67.8–97.5)	77.0 ±9.3 (62.5–104.0)	0.001
Calf circumference			
Rt	34.2 ± 2.8 (28.1–45.0)	32.1 ± 2.6 (26.3–37.5)	<0.001
Lt	34.2 ± 2.8 (27.5–44.0)	32.1 ± 2.5 (26.5–37.3)	<0.001
Comorbidity, mean	1.58 ± 0.97	1.35 ± 1.16	<0.001
None, N (%)	5 (11.1)	12 (26.1)	<0.001
Cardiovascular disease, N (%)	40 (88.9)	29 (63.0)	<0.001
Musculoskeletal disease, N (%)	2 (4.4)	12 (26.1)	<0.001

Mean value ± standard deviation (min–max).

**Table 2:** Comparisons between near-infrared spectroscopy and bioelectrical impedance analysis in the lean mass assessment of older people (N = 91).

Location	Measurements	Mean (Kg)	SD	ICC (2,1)	Cronbach’s Alpha Coefficient
Total	DMW-NIRS	41.94	7.53	0.943	0.949
	BIA	44.18	8.10		
Rt arm	DMW-NIRS	2.30	0.65	0.947	0.950
	BIA	2.36	0.61		
Lt arm	DMW-NIRS	2.10	0.60	0.942	0.944
	BIA	2.34	0.61		
Trunk	DMW-NIRS	21.36	3.36	0.964	0.973
	BIA	19.92	3.80		
Rt leg	DMW-NIRS	6.65	1.43	0.953	0.954
	BIA	6.60	1.49		
Lt leg	DMW-NIRS	6.60	1.35	0.950	0.952
	BIA	6.61	1.55		

DMW-NIRS: discrete multi-wavelength near-infrared spectroscopy; BIA: bioelectrical impedance analysis; SD: standard deviation; ICC: intraclass correlation coefficient. These analyses were performed after log transformation considering the normality of the variables.

**Table 3:** Correlation between calf circumference and muscle mass measured by DMW-NIRS.

Muscle Mass	Total		Men		Women	
	Coefficient	*p*-Value	Coefficient	*p*-Value	Coefficient	*p*-Value
Calf Circumference, Rt						
Total lean mass	0.592	<0.001	0.481	0.001	0.572	<0.001
Rt arm	0.525	<0.001	0.379	0.010	0.520	<0.001
Lt arm	0.577	<0.001	0.425	0.004	0.578	<0.001
Trunk	0.613	<0.001	0.537	<0.001	0.578	<0.001
Rt leg	0.571	<0.001	0.409	0.005	0.542	<0.001
Lt leg	0.566	<0.001	0.363	0.014	0.564	<0.001
Calf Circumference, Lt						
Total lean mass	0.614	<0.001	0.517	<0.001	0.644	<0.001
Rt arm	0.538	<0.001	0.429	0.003	0.570	<0.001
Lt arm	0.592	<0.001	0.470	0.001	0.619	<0.001
Trunk	0.637	<0.001	0.572	<0.001	0.649	<0.001
Rt leg	0.593	<0.001	0.426	0.004	0.623	<0.001
Lt leg	0.595	<0.001	0.408	0.005	0.641	<0.001

Pearson’s correlation analysis was assessed. These analyses were performed after log transformation considering the normality of the variables.

## Data Availability

The data presented in this study are available upon request from the corresponding author.
